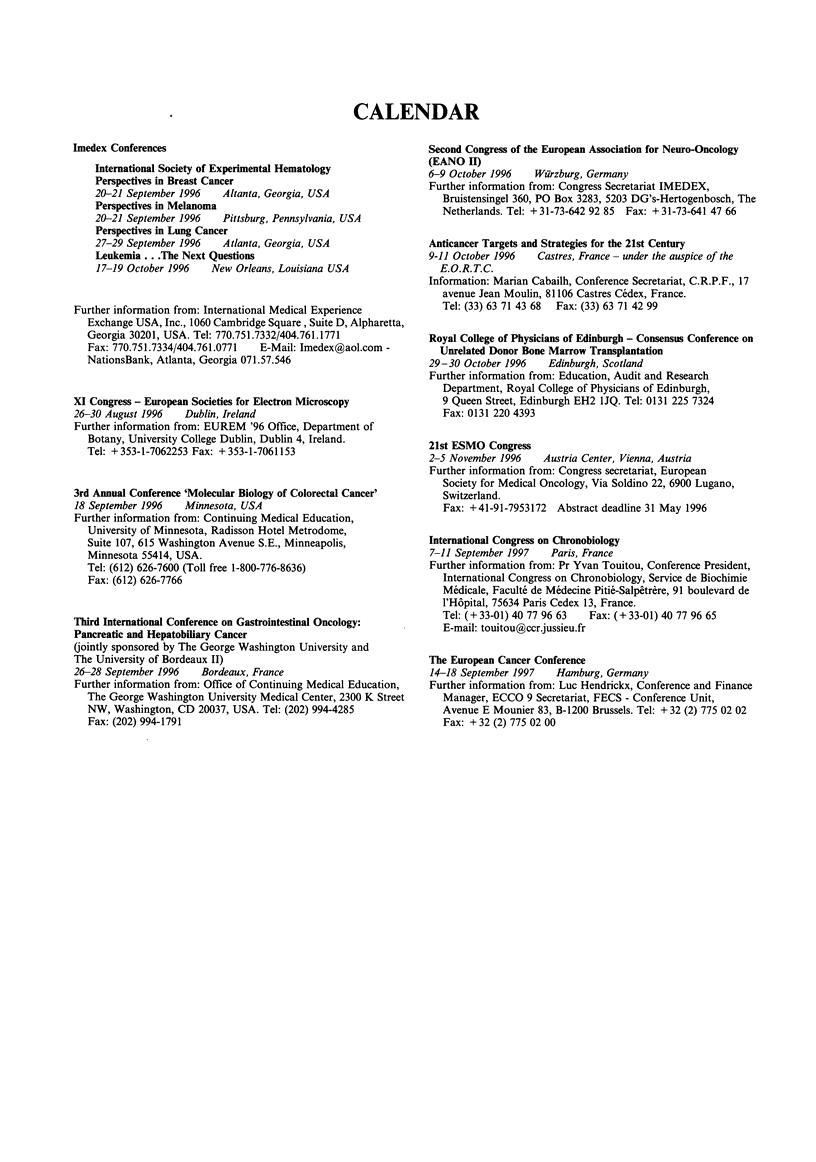# Calendar

**Published:** 1996-09

**Authors:** 


### CALENDAR

**Imedex Conferences**

**International Society of Experimental Hematology**
**Perspectives in Breast Cancer**
*20–21 September 1996 Altanta, Georgia, USA*
**Perspectives in Melanoma**
*20–21 September 1996 Pittsburg, Pennsylvania, USA*
**Perspectives in Lung Cancer**
*27–29 September 1996 Atlanta, Georgia, USA*
**Leukemia . . .The Next Questions**
*17–19 October 1996 New Orleans, Louisiana USA*

Further information from: International Medical Experience Exchange USA, Inc., 1060 Cambridge Square , Suite D, Alpharetta, Georgia 30201, USA. Tel: 770.751.7332/404.761.1771 Fax: 770.751.7334/404.761.0771 E-Mail: Imedex@aol.com - NationsBank, Atlanta, Georgia 071.57.546

**XI Congress – European Societies for Electron Microscopy**
*26–30 August 1996 Dublin, Ireland*
Further information from: EUREM '96 Office, Department of Botany, University College Dublin, Dublin 4, Ireland. Tel: +353-1-7062253 Fax: +353-1-7061153

**3rd Annual Conference 'Molecular Biology of Colorectal Cancer'**
*18 September 1996 Minnesota, USA*
Further information from: Continuing Medical Education, University of Minnesota, Radisson Hotel Metrodome, Suite 107, 615 Washington Avenue S.E., Minneapolis, Minnesota 55414, USA.
Tel: (612) 626-7600 (Toll free 1-800-776-8636)
Fax: (612) 626-7766

**Third International Conference on Gastrointestinal Oncology: Pancreatic and Hepatobiliary Cancer**
(jointly sponsored by The George Washington University and The University of Bordeaux II)
*26–28 September 1996 Bordeaux, France*
Further information from: Office of Continuing Medical Education, The George Washington University Medical Center, 2300 K Street NW, Washington, CD 20037, USA. Tel: (202) 994-4285
Fax: (202) 994-1791

**Second Congress of the European Association for Neuro-Oncology (EANO II)**
*6–9 October 1996 Würzburg, Germany*
Further information from: Congress Secretariat IMEDEX, Bruistensingel 360, PO Box 3283, 5203 DG's-Hertogenbosch, The Netherlands. Tel: +31-73-642 92 85 Fax: +31-73-641 47 66

**Anticancer Targets and Strategies for the 21st Century**
*9-11 October 1996 Castres, France – under the auspice of the E.O.R.T.C.*
Information: Marian Cabailh, Conference Secretariat, C.R.P.F., 17 avenue Jean Moulin, 81106 Castres Cédex, France.
Tel: (33) 63 71 43 68 Fax: (33) 63 71 42 99

**Royal College of Physicians of Edinburgh – Consensus Conference on Unrelated Donor Bone Marrow Transplantation**
*29–30 October 1996 Edinburgh, Scotland*
Further information from: Education, Audit and Research Department, Royal College of Physicians of Edinburgh, 9 Queen Street, Edinburgh EH2 1JQ. Tel: 0131 225 7324
Fax: 0131 220 4393

**21st ESMO Congress**
*2–5 November 1996 Austria Center, Vienna, Austria*
Further information from: Congress secretariat, European Society for Medical Oncology, Via Soldino 22, 6900 Lugano, Switzerland.
Fax: +41-91-7953172 Abstract deadline 31 May 1996

**International Congress on Chronobiology**
*7–11 September 1997 Paris, France*
Further information from: Pr Yvan Touitou, Conference President, International Congress on Chronobiology, Service de Biochimie Médicale, Faculté de Médecine Pitié-Salpêtrère, 91 boulevard de l'Hôpital, 75634 Paris Cedex 13, France.
Tel: (+33-01) 40 77 96 63 Fax: (+33-01) 40 77 96 65
E-mail: touitou@ccr.jussieu.fr

**The European Cancer Conference**
*14–18 September 1997 Hamburg, Germany*
Further information from: Luc Hendrickx, Conference and Finance Manager, ECCO 9 Secretariat, FECS - Conference Unit, Avenue E Mounier 83, B-1200 Brussels. Tel: +32 (2) 775 02 02
Fax: +32 (2) 775 02 00